# Disrupted Circadian Rest-Activity Cycles in Inflammatory Bowel Disease Are Associated With Aggressive Disease Phenotype, Subclinical Inflammation, and Dysbiosis

**DOI:** 10.3389/fmed.2021.770491

**Published:** 2022-02-04

**Authors:** Garth R. Swanson, Nicole Kochman, Jaimin Amin, Vijit Chouhan, Wesley Yim, Phillip A. Engen, Maliha Shaikh, Ankur Naqib, Laura Tran, Robin M. Voigt, Christopher B. Forsyth, Stefan J. Green, Ali Keshavarzian

**Affiliations:** ^1^Division of Digestive Diseases and Nutrition, Department of Internal Medicine, Rush University Medical Center, Chicago, IL, United States; ^2^Rush Medical College, Rush Center for Integrated Microbiome and Chronobiology Research, Rush University Medical Center, Chicago, IL, United States; ^3^Genomics and Microbiome Core Facility, Rush University, Chicago, IL, United States

**Keywords:** inflammatory bowel disease, circadian, rest-activity rhythms, intestinal permeability, microbiota, Crohn's disease, ulcerative colitis, wrist actigraphy

## Abstract

Patients with inflammatory bowel disease (IBD)—Crohn's disease (CD), and ulcerative colitis (UC), have poor sleep quality. Sleep and multiple immunologic and gastrointestinal processes in the body are orchestrated by the circadian clock, and we recently reported that a later category or chronotype of the circadian clock was associated with worse IBD specific outcomes. The goal of this study was to determine if circadian misalignment by rest-activity cycles is associated with markers of aggressive disease, subclinical inflammation, and dysbiosis in IBD. A total of 42 patients with inactive but biopsy-proven CD or UC and 10 healthy controls participated in this prospective cohort study. Subjects were defined as having an aggressive IBD disease history (steroid dependence, use of biologic or immunomodulator, and/or surgery) or non-aggressive history. All participants did two weeks of wrist actigraphy, followed by measurement of intestinal permeability and stool microbiota. Wrist actigraphy was used to calculate circadian markers of rest-activity– interdaily stability (IS), intradaily variability (IV), and relative amplitude (RA). Aggressive IBD history was associated with decrease rest-activity stability (IS) and increased fragmentation compared to non-aggressive IBD and health controls at 0.39 ±.15 vs. 0.51 ± 0.10 vs. 0.55 ± 0.09 (*P* < 0.05) and 0.83 ± 0.20 vs. 0.72 ± 0.14 (*P* < 0.05) but not HC at 0.72 ± 0.14 (*P* = 0.08); respectively. There was not a significant difference in RA by IBD disease history. Increased intestinal permeability and increased TNF-α levels correlated with an increased rest activity fragmentation (IV) at *R* = 0.35, *P* < 0.05 and *R* = 0.37, *P* < 0.05, respectively; and decreased rest-activity amplitude (RA) was associated with increased stool calprotectin at *R* = 0.40, *P* < 0.05. Analysis of intestinal microbiota showed a significant decrease in commensal butyrate producing taxa and increased pro-inflammatory bacteria with disrupted rest-activity cycles. In this study, different components of circadian misalignment by rest-activity cycles were associated with a more aggressive IBD disease history, increased intestinal permeability, stool calprotectin, increased pro-inflammatory cytokines, and dysbiosis. Wrist activity allows for an easy non-invasive assessment of circadian activity which may be an important biomarker of inflammation in IB.

## Introduction

Crohn's disease (CD) and ulcerative colitis (UC) are two forms of inflammatory bowel disease (IBD) characterized by chronic inflammation of the gastrointestinal (GI) tract and recurrent relapses and remissions throughout their course. It is currently estimated that as many as 1.4 million people in the United States and 2.2 million people in Europe suffer from IBD (1, 2). It is, therefore, a significant healthcare burden. It is estimated that the annual disease-attributable direct costs of IBD in the United States is $6.3 billion ($3.6 billion for CD, $2.7 billion for UC) (3, 4). The predominant symptoms of an active disease include diarrhea, abdominal pain, unintentional weight loss, and extraintestinal manifestations including fevers, joint pains, and rashes. In addition, during active disease, symptoms can cause significant disruption to a patient's quality of life (5, 6). Therefore, strategies to prevent flare and retain remission will have a major positive impact on patient quality-of-life (7) and decrease the risk of disease related complications like hospitalization, surgery, and cancer (8, 9).

Although the etiology of IBD and trigger(s) for IBD flare are unknown, clearly important mechanisms include mucosal immune dysregulation, alterations in gut microbiota, and disruptions in the intestinal barrier function (10–12); Therefore, factors that promote gut barrier dysfunction, cause disruption of the microbiota community, and/or drive immune inflammation that could trigger IBD flare. Indeed, epidemiological studies have identified several risk factors such as diet (13), stress (14), medications (15), and infection (16) for disease flare. Furthermore, when IBD is inactive increased intestinal permeability (17, 18), sub-clinical inflammation [increased stool calprotecin (19, 20) and/or inflammatory cytokines (21, 22)] and dysbiosis (23) are predictors of disease flare. However, in spite of advances in medical therapy with biologics and small molecule drugs, a large percentage of patients with IBD still suffer from recurrent flares. Therefore, there is still an unmet need to identify additional environmental factors that could impact subclinical inflammation in IBD and lead to novel therapeutic strategies to prevent flare.

One such potential factor for disease flare that has been increasingly recognized as important in IBD is sleep. Our group (24–26) and others (27, 28), have established that patients with IBD exhibit poor sleep patterns even when their disease is inactive, and at least, patients with CD and inactive disease but poor sleep are at increased risk for mucosal inflammation and disease flare. This makes improving sleep an attractive target in IBD. The sleep-wake cycle and many other critical biological processes including metabolism and immune function are regulated by the circadian clock (29–31), which is a 24-h internal biological system. The central circadian clock, located in the suprachiasmatic nucleus (SCN), is the master clock that entrains the host to the environment via light/dark cycles.

Studies have demonstrated the critical importance of the circadian clock in the regulation of physiology and biology (32, 33). Circadian misalignment occurs when there is a lack of synchronization between the internal circadian clocks and external environmental stimuli. In modern society, circadian misalignment is common with exposure to light at night, long distance travel/jet lag, and shift work. Shift work, which currently makes up ~30% of the US work force, is associated with a variety of metabolic and gastrointestinal tract (GIT) diseases including diabetes (34), peptic ulcer disease (35), colon cancer (36), and irritable bowel syndrome (37). In humans, shift work increases susceptibility of the colonic barrier to an injurious agent like alcohol and increases systemic pro-inflammatory cytokines IL-6 and IL-1β (38). Similarly, chronic alterations in light: dark cycles to induce central circadian misalignment in mouse models of IBD increases colitis severity and mortality compared to animals with normal circadian rhythms (39, 40). In humans with IBD, our group has shown that a later chronotype and increased social jet lag (marker of circadian misalignment) are more common in IBD and associated with worse IBD disease course (41).

Despite the increasing evidence that disruption of circadian rhythms can exacerbate a pro-inflammatory state and could worsen IBD disease course, the prevalence and impact of circadian misalignment in IBD course and severity has not been well-studied. This is in part due to challenges of measuring circadian rhythms in humans. The gold standard of endogenous circadian phase is measured by hourly plasma melatonin (dim light melatonin onset, DLMO) (42). However, this method is both cumbersome and labor intensive, as it must be assessed in the lab under constant conditions with frequent sampling. Therefore, recently there has been an increase in other measures related to circadian rhythms in humans such as rest-activity rhythms (RARs) using wrist actigraphy (43). Wrist actigraphy is a well-validated methodology for objectively measuring sleep (44), but circadian RARs has now been validated in several diseases as a biomarker of circadian misalignment related to a biological impact (45–47).

The goals of this study were to: (1) objectively determine if disruption of circadian RARs (by wrist actigraphy) is common in inactive IBD with an aggressive phenotype; and (2) determine if markers of subclinical inflammation (e.g., intestinal permeability, dysbiosis, inflammatory cytokines) in inactive IBD subjects are associated with objective disruption of circadian RARs.

## Materials and Methods

### Participants

This was a single center prospective cross-over trial involving 47 subjects with an established diagnosis of IBD. All subjects who met the inclusion criteria were recruited from the IBD GI clinic at the Rush University Medical Center. The inclusion criteria for IBD participants included: (1) ≥18 years older; (2) having endoscopy and biopsy proven Crohn's disease or Ulcerative Colitis; (3) having inactive disease defined as a Harvey Bradshaw Index (HBI) <5 for CD (48), or a modified HBI <5 for UC (49). The inclusion criteria for Healthy controls included: (1) ≥18 years older; (2) no history of GI disease including IBS by Rome IV criteria Subjects were excluded if they met any of the following exclusion criteria: (1) unable to give informed consent; (2) prednisone use in the past 4 weeks; (3) antibiotic use within the last 4 weeks; (4) indeterminate colitis; (5) significant chronic organ disease such as: (a) liver disease (AST/ALT >1.5x ULN in the last 12 months), (b) kidney disease (creatinine >1.2 mg/dL in the last 12 months), (c) clinically significant lung disease or heart failure, (d) HIV infection, (e) diabetes, (6) major depression (score >15 on Beck Depression Inventory), (7) sleep apnea (score high risk 2 or more categories on the Berlin Questionnaire), (8) restless leg syndrome [using the International Restless Legs Syndrome Study Group (IRLSSG) consensus criteria for restless leg syndrome], (9) currently taking sedatives or hypnotics, (10) surgical history of ileostomy or colectomy with ileal pouch, (11) night shift work (3 or more nights/week) or more than two time zones crossed in the past 4 weeks, (12) any children <2 years old living at home, (13) currently pregnant, and (14) had any wrist mobility limitations.

All subjects enrolled in the study provided written informed consent at the time of recruitment, and the study and all procedures were approved by the Rush University Medical Center Institutional Review Board. The study was registered on ClinicalTrails.gov- NCT04637399.

### IBD Disease Phenotype

All subjects were categorized by the PI in the Montreal Classification (50) after recruitment, and categorized as “aggressive disease” if they included any of the following criteria: (a) history of steroid dependence (continuous prednisone use for >3 months), (b) history of Crohn's related surgery, (c) use of biologic or immunomodulator agents, or (d) perianal disease in CD. Disease history that did not fit any of these categories on the aggressiveness checklist were classified as “non-aggressive disease.” These definitions were based on previous published data (51–54).

### Questionnaires

All IBD subjects completed several questionnaires including the HBI for CD or modified HBI for UC, and a demographics form. The HBI determines quality of life by assessing the subject's sense of well-being and disease symptoms over the past day, and a score >5 indicates active disease of varying severity. The demographics form collected information regarding subject age, race, gender, past medical history, use of IBD specific medications, past surgical history, and social history. All subjects completed the Beck Depression Inventory, Berlin Questionnaire, and the International Restless Legs Syndrome Study Group (IRLSSG) consensus criteria questionnaire to evaluate severe depression, obstructive sleep apnea, and restless leg syndrome. These questionnaires were utilized to determine if subjects had an underlying condition which would exclude them from the study.

### Circadian Rest-Activity Rhythms

All subjects wore a wrist actigraphy monitor (Philips Spectrum, 30 s epochs) on their non-dominant hand for 14 days. Actigraphy monitoring allows for assessment of gross motor activity in order to establish defined rest-activity cycles. Frequently, a cosine function in relation to biological variables, like core body temperature, is used in the study of circadian rhythmicity which is a parametric analysis (55). The RARs are a biological rhythm that does not follow a sinusoidal waveform, and for this reason other non-parametric variables have been proposed (56). NparACT (R v. 3.8.1) is a validated measure of rest-activity cycles by a non-parametric statistical package (57). It calculates three distinct variables each informing on different aspects of a circadian rhythm. Interdaily stability (IS) is the measure of circadian rhythmicity consistency. The strength of the circadian activity from one day to the next, derived from the 24-h value from a chi-square periodogram, will range from 0 to 1, with a higher value indicating a stronger stability of the rhythm. Thus, it provides information on the degree to which the circadian rhythm is coupled to *zeitgebers*. Again, an IS value closer to 1 is indicative of a stronger coupling to *zeitgebers* within a period of 24 h. Whereas, IS measures rhythmic stability, intradaily variability (IV) measures disturbances in circadian rhythmicity; circadian rhythm fragmentation. Scores range from 0 to 2 with values approaching 2 representing a stronger degree to which active and resting states of a circadian rhythm are disrupted. Though activity levels are expected to vary throughout the day, a healthful circadian rhythm is characterized by one prolonged period of activity followed by one prolonged period of rest. Relative Amplitude (RA) is a value derived from the averaged values of the 5 least active hours (L5) over the course of experiment and the 10 most active hours (M10) over the course of the experiment. The L5 and M10 values inform on the restfulness of a defined rest period and the degree of activeness during defined periods of activity, respectively. RA values range from 0 to 1 with values approaching 1 indicating a greater amplitude difference in rest and active periods, thought to be a robust indicator of circadian rhythm health. Finally, to validate the viability of actigraphy data in measuring the extent to which a circadian rhythm may be disrupted, the variables derived from this methodology were both compared to patient reported data from sleep logs. If participants took off the device, they entered the date and duration into a protocol, and these times were later marked as missing data.

### Intestinal Permeability Measurement

Ingestion of sugar probes, or large and difficult to absorb sugars, is a common method to measure intestinal permeability *in vivo* that has been previously used in humans (58). After a 4-h fast, each subject was asked to empty their bladder completely and each subject ingested 300 ml of liquid containing 7.5 g of lactulose, 2 g of mannitol, 40 g of sucrose, and 2 g of sucralose. Thereafter, all urine produced was collected over 24 h in the following batches: the first 5 h, the next 7 h, and the last 12 h. Subjects were not allowed to eat for 4 h after the start of the urine collection. Urine volumes in each batch were recorded and aliquots of urine were stored at −80°C until analysis. Measurement of urinary sugars was done by gas chromatography and calculated as percent excretion of oral intake. A 5-h urinary sucrose excretion is primarily a marker of gastroduodenal permeability; 5-h urinary lactulose, mannitol, and lactulose/mannitol ratio (L/M) are primarily markers of small bowel permeability, and 24-h urinary sucralose and lactulose excretion are markers of total gut permeability with sucralose primarily representing colonic permeability (59). This is due to both sucralose and lactulose being able to permeate through both the small and large intestine (colon). However, sucralose is not fermented by colonic bacteria while ~75% of lactulose and mannitol are fermented by colonic bacteria (60).

### Inflammatory Cytokine Measurement

IL6 and TNF-α ELISA were done correspondently on human serum using IL-6 ELISA kit (HS600B; R&D systems, Minneapolis, MN, USA) and TNF-α ELISA kit (HSTA00E; R&D systems, Minneapolis, MN, USA).

### Fecal Collection and Calprotectin

Fecal collection fecal samples were self-collected at home, using an anaerobic home collection kit (BD Gaspak, Becton, Dickinson and Company, Sparks, MD, USA). Fecal samples were frozen at the time of collection until it was brought to the RUMC GI Laboratory. Fecal samples were stored at −80°C until analysis. Fecal samples were only subjected to a single freeze-thaw cycle. Calprotectin was done on human stool using ELISA kit (EK-Cal; Buehlmann).

### Fecal Microbiota Interrogation & Analysis

Fecal microbiota was assessed by non-targeted shotgun metagenome sequencing and taxonomic and functional gene profiling. Total DNA was extracted from fecal samples utilizing the FastDNA bead-beating Spin Kit for Soil (MP Biomedicals, Solon, OH, USA), and verified with fluorometric quantitation (Qubit 3.0, Life Technologies, Grand Island, NY, USA). Library preparation was performed using the Celero DNA-Seq library preparation protocol (Nugen, Redwood City, CA) according to the manufacturer's instructions. Library was screened initially using an Illumina MiniSeq mid-output flow cell, and re-pooled and sequenced on an Illumina HiSeq4000 instrument. Library preparation was performed at the Genome Research Core (GRC) at the University of Illinois at Chicago (UIC), and HiSeq4000 sequencing was performed by Novogene Corp (Chula Vista, CA). Raw sequence data (FASTQ files) were deposited in the National Center for Biotechnology Information (NCBI) Sequence Read Archive (SRA), under the BioProject identifier PRJNA576887.

Raw reads were mapped to the NCBI nucleotide database using centrifuge (61, 62). A least common ancestor algorithm was used to determine the taxonomic annotations for each read. The taxonomic annotations were summarized across all reads to create counts per taxon. Raw counts were normalized to percentages for relative abundance. Each fecal sample was rarefied (bacteria: 1.6 million sequences/sample).

Raw reads were aligned to the SwissProt protein database using DIAMOND (63, 64). Functional gene annotations for each read were obtained using a consensus algorithm and then summarized across all reads to create counts per orthologous ID. Higher level summaries of orthologous functions are created using KEGG module, pathway, and BRITE hierarchical annotations (65). Raw counts were normalized to percentages for relative abundance.

### Statistical Analyses

Fecal microbiome was assessed for alterations in the microbial diversity, bacterial community structure and functional gene content. Alpha-diversity metrics [i.e., Shannon's Index, Simpson's index, observed species (richness), and Pielou's evenness] were calculated from rarefied dataset (1.6 million sequences/sample) at the taxonomic level of species. Differences in alpha diversity indices between groups were assessed for significance using one-way analysis of variance (ANOVA) tests with Tukey's *post-hoc* test with adjusted *P*-values reported.

To examine the differences in microbial community structure between samples, pairwise Bray-Curtis dissimilarity (non-phylogenetic) metric was generated using the Primer 7 software package (Primer-E, Ltd. Lutton, UK), and analysis of similarity (ANOSIM) calculations were performed on pair-wise distance matrices to determine if differences in microbial community structure between groups were significant. ANOSIM was performed at the taxonomic level of species on square-root transformed data with 9,999 permutations. Random forest (RF) models (number of runs = 1,023) were used to predict the species-level bacterial profiles, of the three circadian wrist actigraphy alignments, using the R implementation of the algorithm (Boruta algorithm, “RandomForest” package) (66).

To find which individual taxa were most likely to explain the differences between the circadian wrist actigraphy IBD types and alignments sub-groups, the Linear discriminant analysis Effect Size (LEfSE) algorithm was performed (67). Specifically, LEfSE uses the non-parametric factorial Kruskal-Wallis sum-rank test to detect individual taxa differed between the circadian wrist actigraphy IBD types and biological significance is subsequently investigated using a set of pairwise tests among subclasses using the (unpaired) Wilcoxon rank-sum test. As a last step, LEfSe uses Linear Discriminant Analysis to estimate the effect size of each differentially abundant taxa. Differentially abundant taxa that were statistically significant using an alpha of (0.05) and exceeded an LDA log score of at least (± 2) were visually represented in bar plots.

A network analysis was generated based off the Pearson's correlation values using the graph package within the R programming language (68). Pearson's correlations were generated between the relative abundances of bacterial species with IBD clinical and experimental parameters using a significant threshold of *P*-value: (*P* < 0.05) and R value: (>0.3). Functional gene content profiling differential abundances were determined using the software program Statistical Analysis of Metagenomics Profiles (STAMP) version 2.1.3 (69).

Furthermore, chi square tests and *T*-tests were used to compare the two subject groups. Correlation was done by linear regression. Statistical significance was determined by using a *P-*value of <0.05. These statistics were performed using SPSS version 19.0 or R (version 3.8.1) with nParACT package.

## Results

### Baseline Characteristics

**Figure 1:**
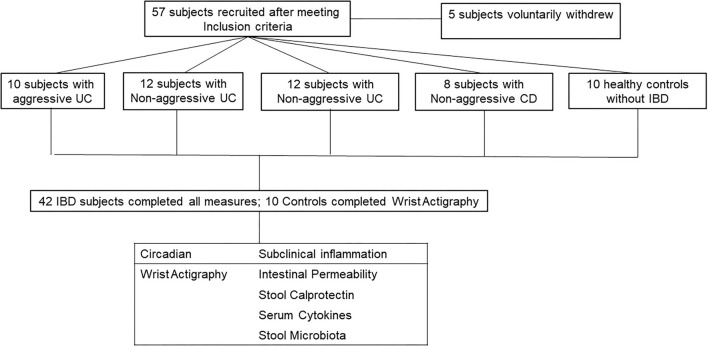
CONSORT flow diagram. 57 subjects were recruited into the study. 5 voluntarily withdrew and did not complete all measures. The 52 subjects who completed all measures, were divided into two categories (Circadian and Subclinical Inflammation). Healthy controls only completed circadian measures as they should have no subclinical inflammation.

A total of 57 subjects met all inclusion/exclusion criteria and thus were enrolled in the study. Five subjects did not complete all portions of the study, three withdrew due to scheduling conflicts, one subject was found to have active disease (HBI >5 at enrollment), and one subject scored high risk in more than two categories of the Berlin questionnaire for sleep apnea. This is summarized in CONSORT flow diagram in Figure 1. A total of 52 subjects completed the study: 10 subjects with aggressive UC, 12 with non-aggressive UC, 12 with aggressive CD, 8 with non-aggressive CD, and 10 healthy controls. Baseline demographics are displayed in Table 1. There were no differences in age, gender, or race between the four groups. The amount of biologic use in the aggressive UC and CD groups was significantly different from subjects with less aggressive disease, consistent with the study design. There was also a higher percentage of pancolitis (E3) in the aggressive UC group and a higher percentage of stricturing or penetrating disease (B2/3) in the aggressive CD group.

**Table 1:** Demographics of IBD subjects at enrollment.

**Disease type**	**UC**	**UC**	**CD**	**CD**	**HC**	***P*-value**
Number of participants	10	12	12	8	10	Total = 52
Aggressive phenotype	Yes	No	Yes	No	N/A	
Mean age	38.3 ± 11.3	41.0 ± 9.8	33.5 ± 7.1	40.7 ± 15.1	34.7 ± 11.2	0.42
**Gender**						
Male	3/10, 30%	4/12, 33%	5/12, 42%	2/8, 25%	2/10, 20%	0.33
Female	7/10, 70%	8/12, 67%	7/12, 58%	6/8, 75%	7/10, 30%	
**Race**						
African American	2/10, 20%	0/12, 0%	1/12, 8%	4/8, 50%	3/10, 30%	0.38
Hispanic or latino	0/10, 0%	2/12, 17%	0/12, 0%	1/8, 12%	1/10, 10%	
Caucasian	8/10, 80%	10/12, 83%	11/12, 92%	3/8, 38%	6/10, 0 60%	
Social history						0.42
Smoker	0/10, 0%	0/12, 0%	2/12, 16%	0/8, 0%	0/10, 0%	
Alcohol use	7/10, 70%	6/12, 50%	11/12, 92%	8/8, 100%	10/10, 100%	
Illicit drug use	0/10, 0%	0/12, 0%	1/12, 8%	0/8, 0%	0/10, 0%	
Biologic/IM use						**<0.05**
Yes	10/10,100%	0/12, 0%	12/12, 100%	0/8, 0%	N/A	
No	0/10, 0%	12/12, 100%	0/12, 0%	8/8, 100%	N/A	
Montreal classification	E3 5/10, 50%	E3 2/12, 17%	B2/3 7/12, 58%	B2/3 0/8, 0%	N/A	

*UC, ulcerative colitis; CD, Crohn's disease; HC, healthy control. Bold values represent a significant difference in biologic use due to the design of the study*.

### Circadian Rest-Activity Rhythms and IBD Disease Course

**Figure 2:**
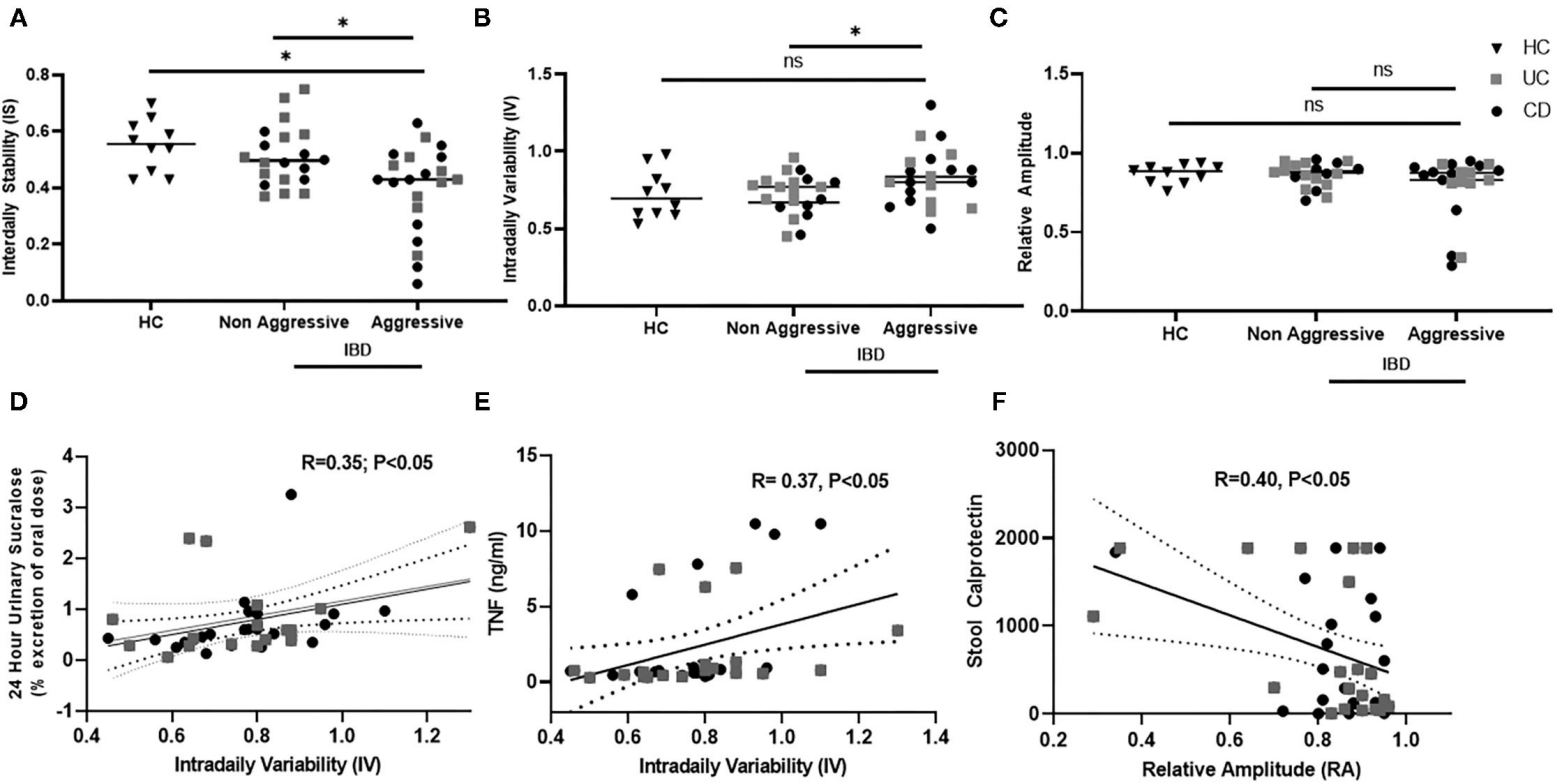
Circadian rest-activity cycles in inactive IBD by disease history and subclinical inflammation. Wrist Actigraphy was used to determine non-parametric variables—interdaily stability (IS), intradaily variability (IV), and relative amplitude (RA). relating to rest-activity circadian cycles. Disease History was compared to **(A)** IS, **(B)** IV, and **(C)** RA. IS and IV were increased in Aggressive IBD. IV associated was associated with increased **(D)** intestinal permeability and **(E)** TNF- α. Decreased RA was associated with **(F)** increased stool calprotectin.

Circadian misalignment was assessed by wrist activity, specifically variables relating to the characteristics of circadian rhythms by wrist actigraphy were calculated by non-parametric analysis—inter-daily stability (IS), intra-daily variability (IV), and relative amplitude (RA). Each of these variables accesses a different aspect of circadian rest-activity cycles as shown in Table 2. Circadian misalignment by wrist actigraphy in IBD with an aggressive and non-aggressive disease course is shown in Figure 2. Decreased IS (stability) were found in IBD with an aggressive disease course compared to non-aggressive disease and healthy controls at 0.39 ± 0.15 vs. 0.51 ± 0.10 vs. 0.55 ± 0.09, (*P* < 0.05); respectively. In addition, increased IV (fragmentation) was found in IBD with an aggressive disease course vs. non-aggressive at 0.83 ± 0.20 vs. 0.72 ± 0.14 (*P* < 0.05); but not HC at 0.72 ± 0.14 (*P* = 0.08); respectively. RA did not vary significantly with IBD disease course or in healthy controls in this cohort at 0.78 ± 0.020 vs. 0.86 ± 0.07 vs. 0.87 ± 0.1 (*P* = 0.63).

**Table 2:** Circadian rest –activity non-parametric measures.

**Variable**	**Measure**	**Circadian misalignment**
IS–interdaily stability	Consistency	Decreased
IV–intradaily variability	Fragmentation	Increased
RA–relative amplitude	Magnitude	Decreased

### Circadian Rest-Activity Rhythms and Markers of Subclinical Inflammation

Circadian rhythms by rest-activity cycles in IBD subjects were then compared to established markers of subclinical inflammation. Data is shown in Figure 2. We found that IV, or rest-activity fragmentation, significantly correlated with increased whole gut permeability assessed by 24-h urinary sucralose (*R* = 0.35, *P* < 0.05). Thus, subjects who had higher permeability had higher fragmentation of their rest-activity cycles. Increased IV was also associated with higher serum TNF levels (*R* = 0.37, *P* < 0.05), indicating increased fragmentation was associated with an increased systemic inflammation. Finally, we found that stool calprotectin, an established marker of intestinal inflammation, was increased in IBD subjects with a decrease in RA (*R* = 0.4, *P* < 0.05). RA is a marker of the amplitude of rest-activity cycles. Thus, intestinal inflammation by stool calprotectin was associated with blunted amplitude of circadian rest-wake activity.

### Circadian Rest-Activity Rhythms and Intestinal Microbiota Structure

Our overall hypothesis was that disruption of rest-activity cycles by wrist actigraphy would be associated with increased pro-inflammatory bacteria, decreased commensal flora, and decreased presence of short chain fatty acid producing bacteria in IBD. To first test this hypothesis, we compared microbial alpha-diversity to circadian outputs assessed *via* wrist actigraphy (i.e., IS, RA, IV). To do so, the non-parametric measure was categorized as either high or low for each group (UC or CD). This analysis revealed that alpha diversity did not differentiate based on circadian wrist actigraphy variables (ANOVA: Supplementary Table 1). Similarly, beta-diversity analyses of the fecal microbial community structures were not different between groups (ANOSIM: Supplementary Table 2).

**Figure 3:**
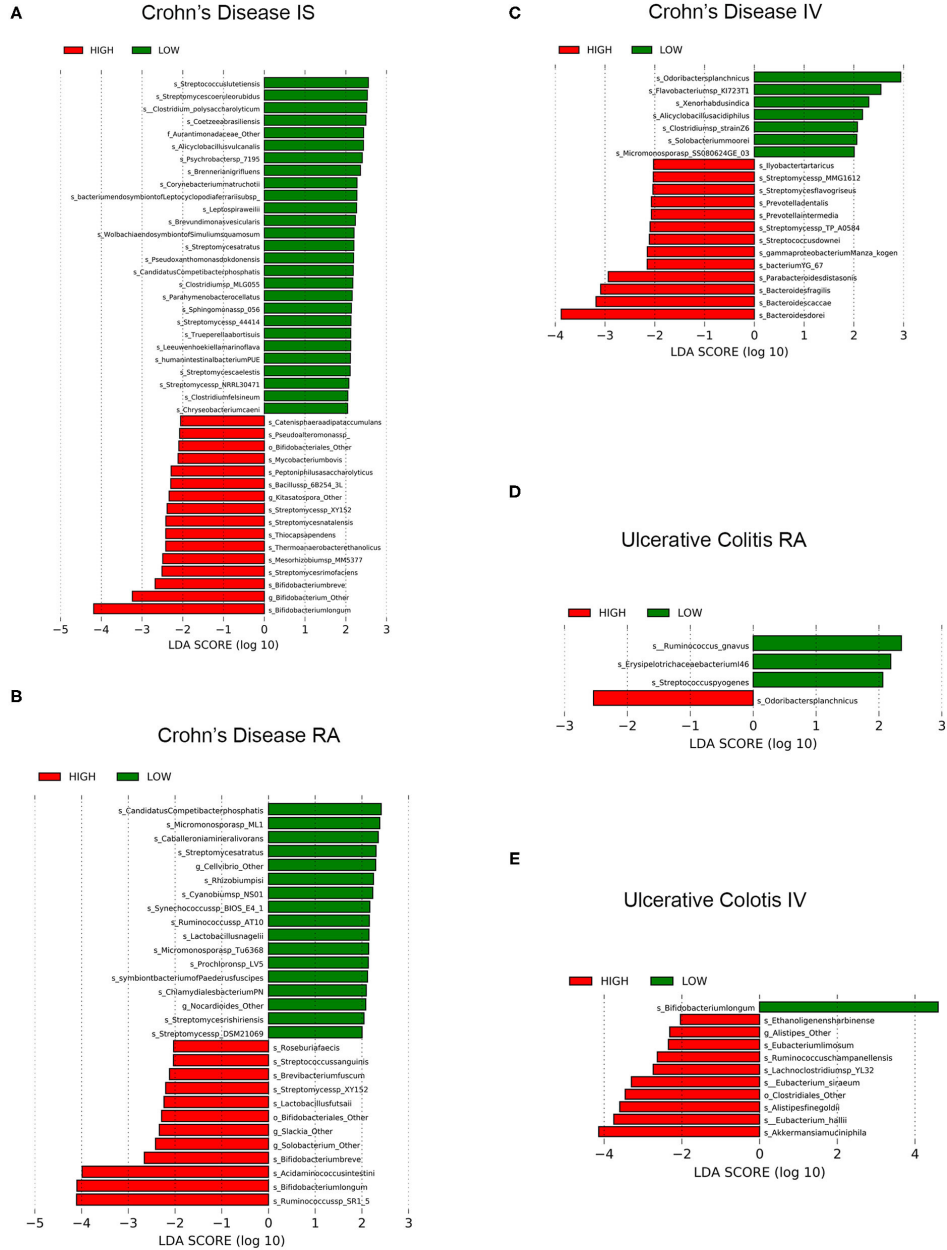
LEfSe analyses of significant fecal microbiomes of Patients with IBD and circadian wrist actigraphy alignments. LEfSe identifies bacterial (species) clades that are differentially abundant within IBD classification types [Crohn's disease (CD) or Ulcerative Colitis (UC)] circadian wrist actigraphy class and alignments subclass. **(A)** CD Interdaily Stability (IS) Low and High **(B)** CD Relative Amplitude (RA) Low and High; **(C)** CD Intradaily Variability (IV) Low and High; **(D)** UC RA; Low and High; and **(E)** UC IV Low and High. Clade colors: Low alignments (green) and High alignments (red). Clades in these graphs were both statistically significantly (*P* < 0.05) and exceeded an LDA log score of at least (± 2).

To more closely examine whether specific taxa were differentially represented in IBD (i.e., CD or UC) vs. rest-activity rhythms (i.e., RA, IV, or IS) status (i.e., high or low) we used the LEfSe algorithm. We found several taxa species to be significantly different in both CD and UC which is summarized below (Figure 3).

### Crohn's Disease

Disruption of rest-activity rhythms by wrist actigraphy significantly impacted stool microbiota community in CD. Changes in microbiota community were characterized by enrichment of putative pro-inflammatory pathobionts and decreased in relative abundance of putative anti-inflammatory bacteria including SCFA producers. Specifically, in CD subjects with low IS (decreased stability of rest-activity rhythms) there was increased putative pathobionts *Streptococcus lutetiensis, S. coeruleorubidus*, and *Clostridium polysaccharolyticum* (70–73), and decreases in 16 taxa species with putative “anti-inflammatory” characteristics were underrepresented [multiple *Bifidobacterium* spp. (74)] (Figure 3A), suggesting a significant loss of commensal beneficial intestinal bacteria.

Crohn's disease (CD) subjects with low RA (low amplitude of rest-activity rhythms) had a total of 17 taxa species that were enriched and the relative abundance of 12 taxa species were decreased including multiple SCFA-producing bacteria: *Ruminococcus* sp SR1/5, *Bifidobacterium* spp., *Acidaminococcus intestine, and Roseburia faecis*) (Figure 3B). These results identify a significant decrease of putative beneficial SCFA-producing intestinal bacteria in CD subjects with low RA.

CD subjects with low IV (decreased fragmentation of rest-activity cycles) compared to low IV, revealed that seven taxa species were enriched including putative SCFA-producing *Odoribacter splanchnicus*, and 13 taxa species were underrepresented which included multiple putative pro-inflammatory bacteria: *Bacteroides* spp., *Streptomyces* spp. and *Prevotella* spp.) (Figure 3C). In conclusion, CD subjects with increased fragmentation of rest-activity rhythms showed microbial communities with a significant increased abundance of multiple pro-inflammatory intestinal bacteria and a decrease in putative SCFA producing bacteria.

A machine-learning algorithm (Boruta) was used to further identify taxa differentiating microbial communities based on circadian alignment. This analysis identified the species *Bifidobacterium* Other and *Bifidobacterium animalis* (IS); Eubacterium eligens and Bifidobacterium longum (RA); and *Bacteroides dorei, Bacteroides fragilis, Odoribacter splanchnicus, Bacteroides* Other, and *Bacteroides helcogenes* (IV) were driving the differences between groups (data not shown). Boruta identifies key species that are different but does not clarify which species are increased or decreased like LEFsE. However, based on the LEFsE data these findings further support our findings of dysbiosis (increased pro-inflammatory and decreased putative bacteria) in CD subjects with disrupted rest-activity rhythms by wrist actigraphy.

### Ulcerative Colitis

Unlike CD, there were no statistically significant differentially abundant taxa species in IS (stability); however, three taxa species were enriched in UC subjects with low RA (amplitude) by rest-activity cycles including *Ruminococcus gnavas* and *Streptococcus pyogenes* (two inflammatory pathobionts), whereas one taxa species was underrepresented (putative SCFA-producing *Odoribacter splanchnicus*) (Figure 3D). Therefore, circadian misalignment in UC is associated with microbial taxa results that indicated significant increased abundance of two pro-inflammatory bacteria and a loss of a putative beneficial SCFA-producing bacteria in UC subjects. Comparing IV (fragmentation) by rest-activity cycles by wrist actigraphy there was enrichment in one taxa species and underrepresentation of 10 taxa, with decreased fragmentation being associated with increased putative beneficial SCFA-producing bacteria (Figure 3E). The differences also showed increased circadian assessment from wrist actigraphy.

Machine learning with Boruta was used to identify important taxa differentiating microbial communities by UC actigraphy alignments. This analysis identified the species *Streptococcus salivarius* and *butyrate producing bacterium* SS3/4 (high IS); *Odoribacter splanchnicus, Blautia obeum* and *Eubacterium siraeum* (high RA); and *Clostridiales* Other, *Parabacteroides* Other, *Akkermansia muciniphila, Alistipes finegoldii, Eubacterium halli*, and *Parabacteroides distasonis* (high IV) were largely driving the differences between circadian alignment groups (data not shown).

In summary, although there were fewer differences in microbiota structure in UC by rest-activity cycles, variables associated with circadian misalignment showed increased pro-inflammatory and decreased putative SCFA producing bacteria.

### Circadian Rest-Activity Rhythms and Intestinal Microbiota Function

Lastly, we investigated the functional gene content profiling of microbiome communities from CD or UC subjects using STAMP which is a software for the Statistical Analysis of Metagenomics Profiles (Supplementary Figure 1). For CD subjects, the non-targeted analysis revealed the abundances of seven genomic pathways for RA (Supplementary Figure 1A), and three genomic pathways for IV (Supplementary Figure 1B). There were no significant functional gene content profile differences noted in CD by IS. Additionally, we explored the functional gene content profiles in UC subjects. The abundances of five genomic pathways by IS: (Supplementary Figure 1C), four genomic pathways by RA (Supplementary Figure 1D), and 21 genomic pathways by IV (Supplementary Figure 1E). Several important metabolic pathways identified that were increased with increased amplitude (RA) of rest-activity cycles in CD include: the anti-inflammatory pentose phosphate pathway mediated by Nrf2 (75), anti-oxidative glucosinolate biosynthesis (76), and amino acids like leucine which are key factors in intestinal homeostasis (77). Similarly, by decreased fragmentation (IV) of rest-activity cycles in UC there was a decrease pentose phosphate pathway and a decreased in the glutathione metabolism which is an important antioxidant in IBD (78).

### Network Analysis of Fecal Microbiome, Circadian Rest-Activity Cycles, and IBD Subjects Variables

To investigate the interaction of clinical and experimental variables like age, gender, IBD types, biological immunomodulatory medication, IBD disease history, rest-activity cycles (assessed via wrist actigraphy), and markers of subclinical inflammatory (intestinal permeability, fecal calprotectin levels, and TNF-α) compared to bacterial taxa. Univariate analysis, using Pearson's correlation, was conducted on the 41 IBD subjects with clinical metadata and microbial taxa at the taxonomic level of species.

This analysis revealed significant correlations between clinical and experimental variables, as well as with bacterial taxa (Pearson: Supplementary Table 3). For example, age positively correlated with *Akkermanisa muciniphila* (*R* = 0.42, *P* = 0.006) and circadian misalignment (fragmentation/IV: *R* = 0.43, *P* = 0.005). Eighteen species were significantly correlated with gender, while IBD type was negatively associated with two putative beneficial butyrate producing species *Faecalibacterium prausnitizi* (*R* = −0.37, *P* = 0.016) and *butyrate-producing bacterium* SS3/4 (*R* = −0.34, *P* = 0.030). Biological immunomodulatory medication positively correlated with TNF-α and history of IBD related surgery, while negatively correlating with circadian misalignment and three *Bacteroides* spp. IBD aggressiveness positively correlated with TNF-α (*R* = 0.50, *P* = 0.002), and negatively correlated with stability of rest-activity cycles (IS: *R* = −0.39, *P* = 0.013) and *butyrate-producing bacterium* SS3/4 (*R* = −0.34, *P* = 0.029). Twenty-five species were significantly correlated with history of IBD related surgery, while history of IBD related surgery was significantly negatively correlated with stability of rest-activity cycles (IS: *R* = −0.49, *P* = 0.007; RA: *R* = −0.47, *P* = 0.010).

When examining the three non-parametric variables of rest-activity cycles by wrist actigraphy, multiple taxa were found to correlate with rest-activity cycles. Examples include: IS (stability) and RA (amplitude) were negatively correlated with *Fusobacterium nucleatum*, bacteria that aggravates colitis in mice (79). Similarly, IS and RA were both negatively correlated with *Escherichia Coli* and *Klebsiella Pneumonia*, both pro-inflammatory pathobiont in IBD (80).

**Figure 4:**
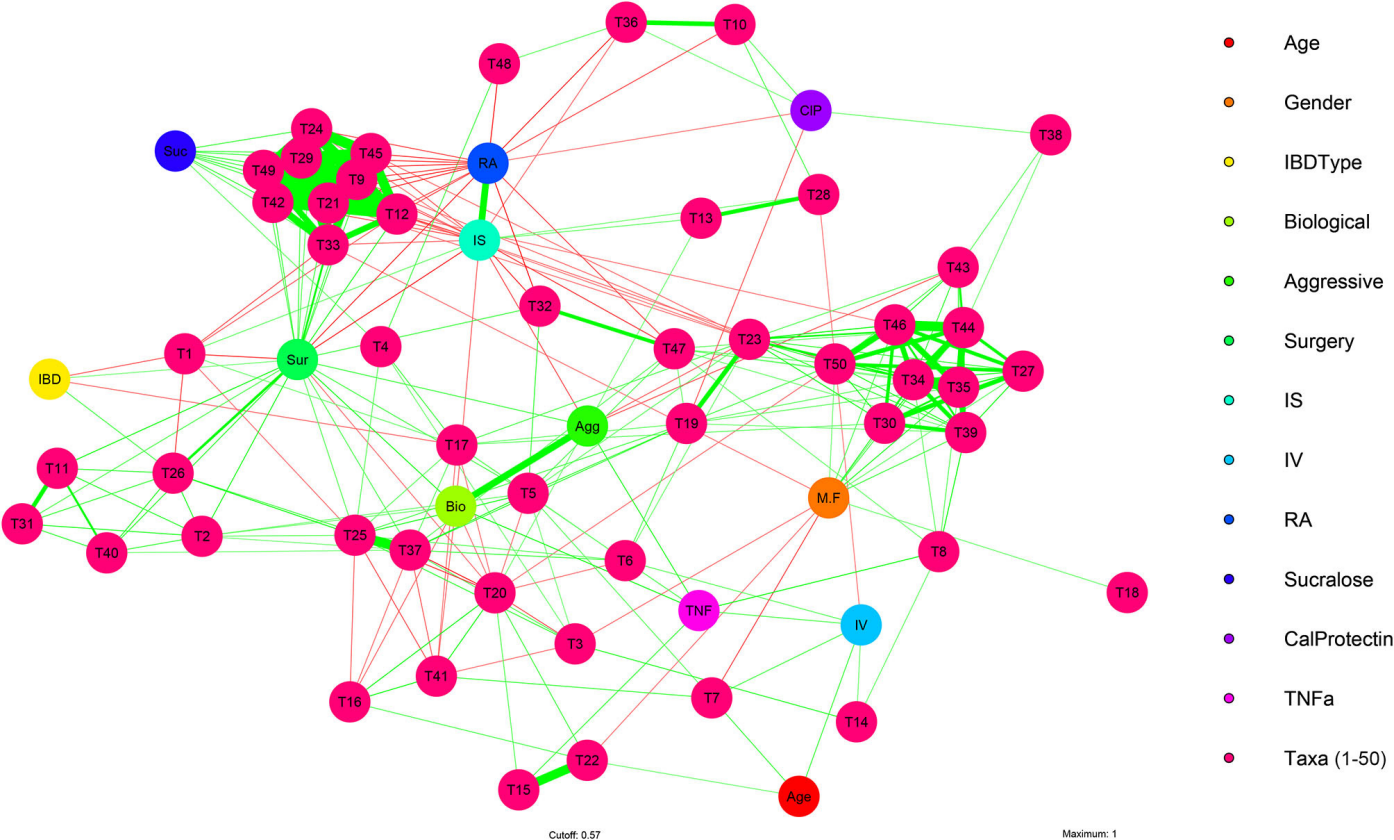
Multivariate analysis of IBD clinical, experimental, and microbiome features. Visualization of multiple significant associations present in clinical and experimental features of IBD subjects with fecal microbiome bacteria (species) using a multivariate network analysis in IBD subjects (*n* = 41). Positive correlations (green arrows), negative correlations (red arrows), strong (thick edges) and weak (thin edges, less saturated) correlations between IBD subject clinical and experimental features, plus bacterial species are shown. Correlation arrows displayed are significant (*P* < 0.05) and have R values >0.30. Reference Supplementary Tables 3, 4 for and statistical data and graphical identifiers.

A multivariate analysis of the microbial features identified above was performed to visualize interactions between the fecal microbiome and the clinical and experimental features at the taxonomic level of species (Figure 4; Supplementary Table 4). This analysis identified correlational clusters (T9–49) between *Escherichia coli, Fusobacterium nucleatum, Shigella sonnei, Klebsiella pneumoniae, Veillonella atypica, Streptococcus lutetiensis, Blautia hansenii, Clostridium perfringens*, and *Clostridium butyricum*, and between (T27–50) *Ruminococcus champanellensis, Oscillibacter valericigenes, Acutalibacter muris, Ruminiclostridium* sp.KB18, *Christensenella massiliensis, Campylobacter jejuni, Ruminococcaceae bacterium* CPB6, *Ethanoligenens harbinense*, and *Ruminococcus albus* (Figure 4; Supplementary Table 4). The stability and amplitude of rest-activity cycles (RA and IS) strongly negatively correlated with the clustering (T9–T49) of microbial species which include several key pro-inflammatory bacteria like *Escherichia coli, Fusobacterium nucleatum, Shigella sonnei, Klebsiella pneumoniae* which have been found to be key taxa in IBD microbiome (81). However, these types of analysis are limited by the overall heterogeneity and temporal instability of the microbiota in IBD.

## Discussion

Our aim was to determine if circadian misalignment by rest-activity assessment is associated with a more aggressive IBD disease phenotype in inactive IBD, and/or presence of subclinical inflammation including intestinal microbiota dysbiosis. To investigate this association, we utilized wrist actigraphy to examine circadian rhythms by rest-activity cycles. We used a non-parametric analysis to examine wrist actigraphy data that examines different characteristics of the rhythm of rest-activity data. Specifically, we calculated three variables which examine different characteristics related to rest-activity: instability (IS), fragmentation (IV), and amplitude (RA), respectively. The main findings of this manuscript are: (1) decreased stability of rest-activity (IS) was associated with an aggressive IBD phenotype; (2) increased fragmentation (IV) and decreased amplitude (RA) correlated with markers of subclinical inflammation (intestinal permeability, increased serum cytokine TNF-α, and stool calprotectin); and (3) “pro-inflammatory” changes in microbial structure and function were associated with disruption of rest-activity which are markers of circadian misalignment. Interestingly, different components of circadian misalignment assessed by rest-activity rhythms (IS, RA, IV) were associated with different IBD-related variables suggesting a dynamic and complex relationship between IBD, inflammation, and circadian machinery that warrants further examination.

These findings support that different components of the circadian system assessed by wrist actigraphy are associated with different disease aspects in IBD. The IV or fragmentation relates to the degeneration of the circadian timing system which is associated with daytime sleepiness or nocturnal arousals, poor sleep, decreased quality of life, poor cognitive and motor performance (56, 82, 83). IS correlates with synchronization of the circadian system with external zeitgebers like light-dark cycles through photic synchronization (84). In this cohort, disease severity was associated strongly with the synchronization circadian timing system (IS) but subclinical inflammation was more sensitive to detect by fragmentation or poor functioning of the circadian timing system (IV).

The results of current study expands upon our prior findings using questionnaires to assess circadian propensity or chronotype which found that a late chronotype and increased social jet lag are associated with a worse disease course in inactive IBD (41), and RARs are associated with GI symptoms in IBD (85). Circadian misalignment is an important possible risk factor for disease flare in IBD as night shift work has been associated with increased inflammation (e.g., salivary TNF-α, IL1β, IL-6) (86). In addition, our group reported that night shift work is associated with increased serum cytokines, decreased resiliency of the colonic barrier, and altered bacterial metabolite profile (38, 87, 88). Therefore, the present study adds to the rationale that circadian misalignment could promote a disease flare in IBD and approaches to stabilize circadian rhythms (e.g., circadian hygiene) might decrease frequency and severity of IBD flare.

Analysis of the intestinal microbiome reveals significant differences in the microbiome communities differ depending on circadian RARs. Increased fragmentation and decreased stability of RARs was associated with an increase in the abundance of pro-inflammatory taxa and reduced abundance of commensal bacteria. Of particular interest is the finding that circadian misalignment defined by rest-activity cycles showed a decrease in short chain fatty acid (SFCA) producing bacteria. SCFA like butyrate are known to have anti-inflammatory properties and a protective effect on the epithelial barrier function through increased intestinal epithelial cell proliferation, mucin production, increases interstitial permeability (76), and bacterial translocation (89). Moreover, butyrate is a well-known predictor of IBD flare and a hallmark of aggressive IBD phenotype (90). Previously, we also found that night shift workers with circadian misalignment had low serum butyrate which correlated with increased colonic permeability (87).

This study does have several important limitations. First, we had a relatively small sample size of 42 participants with inactive IBD and 10 healthy controls which may have limited the power to detect differences. Despite our sample size, we were able to derive significant results regarding primary outcomes; however, further larger scale studies are needed to determine the effect of circadian misalignment on long term clinical efficacy in IBD including disease control and risk for consequences such as surgery and/or hospitalizations. Our study also utilized wrist actigraphy to access RARs which is not the gold standard of circadian timing, instead of DLMO, which is done under controlled laboratory conditions. While our prior work showed correlation with RARs and DLMO (87), RARs are only a biomarker related to circadian misalignment and are an incomplete measure of circadian timing and entrainment. In addition, we used a non-parametric analysis of wrist actigraphy as an objective measurement of RARs and did not use parametric analysis models like cosinor. Non-parametric analysis was used as this methodology does not follow the assumption that the rest-activity behaves similarly to a sinusoidal wave but other methods have been proposed including machine learning (91). Our study also relied on retrospective markers of aggressive disease in IBD and validated non-invasive markers of disease activity like stool calprotectin or intestinal permeability. Future studies should examine the gold standard for central circadian clock measure, dim light melatonin onset, compared to the gold standard of disease activity in IBD, endoscopy and mucosal activity, in a prospective manner under controlled in-lab simulated circadian environment. The results from this study provide a strong scientific rationale that circadian misalignment is a very likely risk factor for IBD flare that should be further examined in prospective clinical trials. Furthermore, wrist actigraphy is also a valuable tool that should be further utilized in future studies and in GI clinics in IBD for objective measurement of sleep and circadian cycles. Addressing circadian rhythms in IBD will advance the goals of personalized medicine and identify modifiable risk factors that can impact the disease course in IBD.

## Data Availability Statement

The original contributions presented in the study are publicly available. This data can be found here: https://www.ncbi.nlm.nih.gov/bioproject/PRJNA576887/.

## Ethics Statement

The studies involving human participants were reviewed and approved by Rush University IRB Board. The patients/participants provided their written informed consent to participate in this study.

## Author Contributions

GS and AK contributed to conception and design of the study. Patients were recruited by NK, JA, VC, and WY. VC and NK organized the database. MS and LT performed assays related to outcomes. PE, AN, and SG analyzed and wrote the microbiome analysis. GS, RV, CF, and AK wrote sections of the manuscript. All authors contributed to the article and approved the submitted version.

## Funding

This work was funded by the National Institutes of Health, R24 AA026801-01 NIH-NIAAA (PI: AK, Co-I: GS) and R01DK124280-01A1 NIH-NIDDK (PI: GS).

## Conflict of Interest

The authors declare that the research was conducted in the absence of any commercial or financial relationships that could be construed as a potential conflict of interest.

## Publisher's Note

All claims expressed in this article are solely those of the authors and do not necessarily represent those of their affiliated organizations, or those of the publisher, the editors and the reviewers. Any product that may be evaluated in this article, or claim that may be made by its manufacturer, is not guaranteed or endorsed by the publisher.

## Abbreviations

IBD: Inflammatory Bowel Disease
CD: Crohn's Disease
UC: Ulcerative Colitis
IS: interdaily stability
IV: intradaily variability
RA: relative amplitude
6-SM: 6-sulfatoxymelatonin
SCN: suprachiasmatic nucleus.
