# Supplementary material for: Disrupted Circadian Rest-Activity Cycles in Inflammatory Bowel Disease Are Associated With Aggressive Disease Phenotype, Subclinical Inflammation, and Dysbiosis
Source: Front Med (Lausanne). 2022 Feb 4;8:770491. doi: 10.3389/fmed.2021.770491 (PMC8900134; doi:10.3389/fmed.2021.770491)
Supplement: Supplementary file 1 [file Table_1.DOCX]

**Supplementary Table 1.** Alpha-diversity values of fecal samples, at the taxonomic level of species, within IBD subject’s circadian wrist actigraphy alignment groups.

| **Alpha Diversity** | **IBD Group** | **IS**  **Mean ± (SD)** | **ANOVA**  ***P*-Value** | **IV**  **Mean ± (SD)** | **ANOVA**  ***P*-Value** | **RA**  **Mean ± (SD)** | **ANOVA**  ***P*-Value** |
| --- | --- | --- | --- | --- | --- | --- | --- |
| Shannon | UC High | 3.83 **±** (0.75) | F (_3, 36_) = 0.3816, *P* = 0.7668 | 4.14 **±** (0.47) | F (_3, 36_) = 1.663, *P* = 0.1921 | 3.99 **±** (0.55) | F (_3, 36_) = 0.1100,  *P* = 0.9537 |
|  | UC Low | 4.01 **±** (0.35) |  | 3.66 **±** (0.62) |  | 3.86 **±** (0.63) |  |
|  | CD High | 4.02 **±** (0.34) |  | 4.01 **±** (0.41) |  | 3.97 **±** (0.31) |  |
|  | CD Low | 3.84 **±** (0.58) |  | 3.87 **±** (0.51) |  | 3.91 **±** (0.59) |  |
|  | | | | | | | |
| Simpson | UC High | 0.89 **±** (0.11) | F (_3, 36_) = 1.270, *P* = 0.2992 | 0.93 **±** (0.02) | F (_3, 36_) = 1.280, *P* = 0.2960 | 0.93 **±** (0.03) | F (_3, 36_) = 0.7844,  *P* = 0.5105 |
|  | UC Low | 0.94 **±** (0.01) |  | 0.89 **±** (0.11) |  | 0.90 **±** (0.11) |  |
|  | CD High | 0.93 **±** (0.02) |  | 0.93 **±** (0.02) |  | 0.94 **±** (0.01) |  |
|  | CD Low | 0.92 **±** (0.05) |  | 0.93 **±** (0.04) |  | 0.92 **±** (0.04) |  |
|  | | | | | | | |
| Observed Species | UC High | 4,382 **±** (270.00) | F (_3, 36_) = 0.2910, *P* = 0.8316 | 4,451 **±** (251.10) | F (_3, 36_) = 1.940, *P* = 0.1406 | 4,351 **±** (290.80) | F (_3, 36_) = 0.9039,  *P* = 0.4488 |
|  | UC Low | 4,272 **±** (236.60) |  | 4,286 **±** (222.40) |  | 4,403 **±** (207.00) |  |
|  | CD High | 4,306 **±** (217.50) |  | 4,389 **±** (256.00) |  | 4,229 **±** (127.30) |  |
|  | CD Low | 4,294 **±** (316.50) |  | 4,213 **±** (242.70) |  | 4,373 **±** (337.60) |  |
|  | | | | | | | |
| Evenness | UC High | 0.46 **±** (0.09) | F (_3, 36_) = 0.4899, *P* = 0.6915 | 0.49 **±** (0.05) | F (_3, 36_) = 1.683, *P* = 0.1878 | 0.48 **±** (0.06) | F (_3, 36_) = 0.1618,  *P* = 0.9213 |
|  | UC Low | 0.48 **±** (0.04) |  | 0.44 **±** (0.07) |  | 0.46 **±** (0.07) |  |
|  | CD High | 0.48 **±** (0.04) |  | 0.48 **±** (0.05) |  | 0.48 **±** (0.04) |  |
|  | CD Low | 0.46 **±** (0.07) |  | 0.46 **±** (0.06) |  | 0.47 **±** (0.07) |  |
| Shannon Index, Simpson’s Index, observed species (richness) and Pielou’s evenness were measured at the taxonomic level of species. Datasets were rarefied to 1,600,000 sequences per sample. Mean index score and standard deviation (SD) are displayed. IS: Interdaily Stability, IV: Intradaily Variability, RA: Relative Amplitude, ANOVA = one-way analysis of variance. There were no significant results for Tukey’s multiple comparisons test. Total of 40 samples (n = 9-11 per group). | | | | | | | |

**Supplementary Table 2.** Group analysis of similarity (ANOSIM) results for overall fecal microbiota community structures, at the taxonomic level of species, between IBD subject’s circadian wrist actigraphy alignment groups.

| **Ulcerative Colitis (UC)** | **R value** | ***P*-value** |
| --- | --- | --- |
|  | | |
| Interdaily Stability (IS) |  |  |
| UC High vs. UC Low | -0.046 | 0.829 |
|  | | |
| Intradaily Variability (IV) |  |  |
| UC High vs. UC Low | 0.093 | 0.069 |
|  | | |
| Relative amplitude (RA) |  |  |
| UC High vs. UC Low | 0.03 | 0.221 |
|  |  |  |
| **Crohn’s Disease (CD)** | **R value** | ***P*-value** |
|  |  |  |
| Interdaily Stability (IS) |  |  |
| CD High vs. CD Low | -0.068 | 0.875 |
|  |  |  |
| Intradaily Variability (IV) |  |  |
| CD High vs. CD Low | -0.027 | 0.624 |
|  |  |  |
| Relative amplitude (RA) |  |  |
| CD High vs. CD Low | -0.026 | 0.584 |
|  |  |  |
| Global *R* comparison was based on ANOSIM performed within the software package Primer7. *P*-values were calculated based on a permutational analysis (9,999 permutations) using square-root transformed data. IS, interdaily stability; IV, intradaily variability; RA, relative amplitude. Total of 40 samples were examined (n = 9-11 per group). | | |

**Supplementary Table 3.** Significant relationships of clinical, experimental and microbiome variable comparisons.

| **Variable 1** | **Variable 2** | **R value** | ***P*-value** |
| --- | --- | --- | --- |
| Age | IV | 0.431 | **0.005** |
| Age | *Akkermansia muciniphila* | 0.420 | **0.006** |
| Age | *Parabacteroides distasonis* | 0.310 | **0.049** |
|  | | | |
| Gender | *Ruminiclostridium* sp.KB18 | 0.478 | **0.002** |
| Gender | *Acutalibacter muris* | 0.477 | **0.002** |
| Gender | *Akkermansia muciniphila* | -0.419 | **0.006** |
| Gender | *Christensenella massiliensis* | 0.409 | **0.008** |
| Gender | *Ethanoligenens harbinense* | 0.388 | **0.012** |
| Gender | *Ruminococcaceae bacterium* CPB6 | 0.387 | **0.012** |
| Gender | *Actinobacteria* Other | 0.372 | **0.017** |
| Gender | *Oscillibacter valericigenes* | 0.346 | **0.027** |
| Gender | *Ruminococcaceae* Other | 0.341 | **0.029** |
| Gender | *Pseudomonas* Other | 0.332 | **0.034** |
| Gender | *Streptomyces* Other | 0.327 | **0.037** |
| Gender | *Ruminococcus champanellensis* | 0.323 | **0.039** |
| Gender | *Proteobacteria* Other | 0.322 | **0.040** |
| Gender | *Eubacterium eligens* | 0.321 | **0.041** |
| Gender | *Ruminococcus* sp.SR1/5 | -0.317 | **0.043** |
| Gender | *Burkholderia* Other | 0.316 | **0.044** |
| Gender | *Ruminococcus albus* | 0.313 | **0.046** |
|  | | | |
| IBD Type | *Lactococcus lactis* | 0.387 | **0.012** |
| IBD Type | *Faecalibacterium prausnitzii* | -0.374 | **0.016** |
| IBD Type | *Butyrate producing bacterium* SS3/4 | -0.339 | **0.030** |
| IBD Type | *Erysipelotrichaceae* Other | 0.311 | **0.048** |
|  |  |  |  |
| Biological | IBD aggressiveness | 0.809 | **1.54019E-10** |
| Biological | TNF-α | 0.550 | **0.0004** |
| Biological | History of IBD related surgery | 0.516 | **0.002** |
| Biological | IS | -0.360 | **0.022** |
| Biological | *Bacteroides uniformis* | -0.338 | **0.030** |
| Biological | *Bacteroides fragilis* | -0.337 | **0.031** |
| Biological | *Eubacterium rectale* | 0.332 | **0.033** |
| Biological | *Bacteroides caecimuris* | -0.319 | **0.041** |
|  | | | |
| Aggressive | TNF-α | 0.491 | **0.002** |
| Aggressive | IS | -0.389 | **0.013** |
| Aggressive | *Campylobacter jejuni* | -0.379 | **0.014** |
| Aggressive | History of IBD related surgery | 0.425 | **0.017** |
| Aggressive | *Butyrate producing bacterium* SM4/1 | -0.342 | **0.029** |
|  | | | |
| Surgery | *Lactococcus lactis* | 0.636 | **0.0001** |
| Surgery | *Clostridium perfringens* | 0.610 | **0.0002** |
| Surgery | *Acidaminococcus intestini* | 0.533 | **0.002** |
| Surgery | *Eubacterium rectale* | 0.508 | **0.004** |
| Surgery | *Blautia hansenii* | 0.496 | **0.005** |
| Surgery | *Faecalibacterium prausnitzii* | -0.484 | **0.006** |
| Surgery | IS | -0.486 | **0.006** |
| Surgery | *Lachnoclostridium* Other | 0.476 | **0.007** |
| Surgery | RA | -0.465 | **0.010** |
| Surgery | *Libanicoccus massiliensis* | 0.446 | **0.012** |
| Surgery | *Fusobacterium nucleatum* | 0.442 | **0.013** |
| Surgery | *Enterobacteriaceae* Other | 0.439 | **0.013** |
| Surgery | *Shigella* Other | 0.438 | **0.014** |
| Surgery | *Streptococcus* Other | 0.433 | **0.015** |
| Surgery | *Clostridium butyricum* | 0.431 | **0.015** |
| Surgery | *Shigella sonnei* | 0.430 | **0.016** |
| Surgery | *Escherichia coli* | 0.425 | **0.017** |
| Surgery | *Streptococcus pasteurianus* | 0.419 | **0.019** |
| Surgery | *Veillonella atypica* | 0.411 | **0.022** |
| Surgery | *Streptococcus lutetiensis* | 0.408 | **0.023** |
| Surgery | *sEubacterium hallii* | 0.393 | **0.029** |
| Surgery | *Lachnoclostridium phytofermentans* | 0.376 | **0.037** |
| Surgery | *Megasphaera elsdenii* | 0.371 | **0.040** |
| Surgery | *Clostridium sphenoides* | 0.369 | **0.041** |
| Surgery | *Klebsiella pneumoniae* | 0.367 | **0.042** |
| Surgery | *Enterobacterales* Other | 0.363 | **0.045** |
| Surgery | *Bacteroidales* Other | -0.359 | **0.047** |
|  | | | |
| IS | RA | 0.805 | **3.65064E-10** |
| IS | *Streptococcus pasteurianus* | -0.460 | **0.003** |
| IS | *Streptococcus suis* | -0.439 | **0.005** |
| IS | *Streptococcus* Other | -0.412 | **0.008** |
| IS | *Fusobacterium nucleatum* | -0.404 | **0.010** |
| IS | *Blautia hansenii* | -0.403 | **0.010** |
| IS | *Clostridium butyricum* | -0.391 | **0.013** |
| IS | *Veillonella atypica* | -0.384 | **0.014** |
| IS | *Streptococcus lutetiensis* | -0.380 | **0.016** |
| IS | *Shigella sonnei* | -0.367 | **0.020** |
| IS | *Shigella* Other | -0.354 | **0.025** |
| IS | *Enterobacteriaceae* Other | -0.350 | **0.027** |
| IS | *Clostridium perfringens* | -0.350 | **0.027** |
| IS | *Escherichia coli* | -0.346 | **0.029** |
| IS | *Lactobacillus gasseri* | -0.345 | **0.029** |
| IS | *Alistipes finegoldii* | 0.342 | **0.031** |
| IS | *Faecalibacterium prausnitzii* | 0.335 | **0.035** |
| IS | *Bifidobacterium animalis* | 0.322 | **0.043** |
| IS | *Parvimonas micra* | -0.320 | **0.044** |
| IS | *Alistipes* Other | 0.315 | **0.048** |
|  | | | |
| IV | *Akkermansia muciniphila* | 0.394 | **0.012** |
| IV | TNF-α | 0.393 | **0.016** |
| IV | *Ruminococcus torques* | 0.356 | **0.024** |
| IV | *Streptococcus thermophilus* | 0.320 | **0.044** |
|  | | | |
| RA | *Streptococcus pasteurianus* | -0.537 | **0.0003** |
| RA | *Streptococcus* Other | -0.511 | **0.001** |
| RA | *Fusobacterium nucleatum* | -0.497 | **0.001** |
| RA | *Lactobacillus gasseri* | -0.488 | **0.001** |
| RA | *Clostridium butyricum* | -0.484 | **0.002** |
| RA | *Parvimonas micra* | -0.478 | **0.002** |
| RA | *Veillonella atypica* | -0.477 | **0.002** |
| RA | *Streptococcus lutetiensis* | -0.473 | **0.002** |
| RA | *Shigella sonnei* | -0.467 | **0.002** |
| RA | *Shigella* Other | -0.457 | **0.003** |
| RA | *Enterobacteriaceae* Other | -0.454 | **0.003** |
| RA | *Escherichia coli* | -0.450 | **0.004** |
| RA | *Klebsiella pneumoniae* | -0.418 | **0.007** |
| RA | *Streptococcus suis* | -0.414 | **0.008** |
| RA | *Enterobacterales* Other | -0.411 | **0.008** |
| RA | *Blautia hansenii* | -0.408 | **0.009** |
| RA | *Clostridium perfringens* | -0.397 | **0.011** |
| RA | *Bifidobacterium adolescentis* | -0.394 | **0.012** |
|  | | | |
| Sucralose | *Enterobacterales* Other | 0.554 | **0.0003** |
| Sucralose | *Enterobacteriaceae* Other | 0.472 | **0.003** |
| Sucralose | *Shigella* Other | 0.469 | **0.003** |
| Sucralose | *Blautia hansenii* | 0.459 | **0.004** |
| Sucralose | *Escherichia coli* | 0.458 | **0.004** |
| Sucralose | *Shigella sonnei* | 0.453 | **0.004** |
| Sucralose | *Klebsiella pneumoniae* | 0.439 | **0.006** |
| Sucralose | *Fusobacterium nucleatum* | 0.433 | **0.007** |
| Sucralose | *Clostridium butyricum* | 0.427 | **0.008** |
| Sucralose | *Streptococcus lutetiensis* | 0.424 | **0.008** |
| Sucralose | *Veillonella atypica* | 0.418 | **0.009** |
| Sucralose | *Streptococcus* Other | 0.408 | **0.011** |
| Sucralose | *Clostridium perfringens* | 0.340 | **0.036** |
| Sucralose | *Roseburia intestinalis* | 0.330 | **0.043** |
|  |  |  |  |
| Calprotectin | *Clostridium saccharolyticum* | -0.376 | **0.020** |
| Calprotectin | *Bifidobacterium adolescentis* | 0.346 | **0.033** |
| Calprotectin | *Enterococcus faecalis* | 0.327 | **0.045** |
|  | | | |
| TNF-α | *Adlercreutzia equolifaciens* | 0.545 | **0.0004** |
| TNF-α | *Eggerthellaceae* Other | 0.529 | **0.001** |
| TNF-α | *Bacteroides cellulosilyticus* | 0.415 | **0.011** |
| TNF-α | *Ruminococcus torques* | 0.373 | **0.023** |
| Pearson correlation (R) values are shown. Only R values of 0.30 or greater, with a corresponding p-value of ˂ 0.05, are depicted. Age: Ages of subjects; Gender: male and female classifications of subjects; IBD Type: Inflammatory Bowel Disease (Ulcerative Colitis and Crohn’s Disease); Biological: biologic immunomodulatory usage of subjects; Aggressive: Inflammatory Bowel Disease treatment aggressiveness scale score of subjects; Surgery: history of Inflammatory Bowel Disease related surgery for subjects; IS: Inter-daily Stability assessment score for subjects; IV: Inter-daily Variability assessment score for subjects; RA: Relative Amplitude assessment score for subjects; Sucralose: sugar measurement in urine at 24 hour period for subjects; Calprotectin: fecal test used to detect inflammation levels in subjects; TNF-α: Tumor necrosis factor-alpha ELISA measurements of subjects. | | | |

**Supplementary Table 4.** Multivariate analysis of microbial taxa identifiers.

| **Taxa Number** | **Taxonomic Level of Species** |
| --- | --- |
| T1 | *Faecalibacterium prausnitzii* |
| T2 | *[Eubacterium] rectale* |
| T3 | *Ruminococcus sp.SR1/5* |
| T4 | *Roseburia intestinalis* |
| T5 | *[Eubacterium] hallii* |
| T6 | *[Ruminococcus] torques* |
| T7 | *Akkermansia muciniphila* |
| T8 | *Adlercreutzia equolifaciens* |
| T9 | *Escherichia coli* |
| T10 | *Bifidobacterium adolescentis* |
| T11 | *Acidaminococcus intestini* |
| T12 | *Blautia hansenii* |
| T13 | *Alistipe finegoldii* |
| T14 | *Streptococcus thermophilus* |
| T15 | *Bacteroides cellulosilyticus* |
| T16 | *Bacteroides fragilis* |
| T17 | *butyrate-producing bacterium SS3/4* |
| T18 | *[Eubacterium] eligens* |
| T19 | *[Clostridium] saccharolyticum* |
| T20 | *Bacteroides caecimuris* |
| T21 | *Veillonella atypica* |
| T22 | *Parabacteroides distasonis* |
| T23 | *butyrate-producing bacterium SM4/1* |
| T24 | *Klebsiella pneumoniae* |
| T25 | *[Clostridium] sphenoides* |
| T26 | *Lactococcus lactis* |
| T27 | *Ruminococcus champanellensis* |
| T28 | *Bifidobacterium animalis* |
| T29 | *Clostridium butyricum* |
| T30 | *Oscillibacter valericigenes* |
| T31 | *Megasphaera elsdenii* |
| T32 | *Streptococcus pasteurianus* |
| T33 | *Clostridium perfringens* |
| T34 | *Acutalibacter muris* |
| T35 | *Ruminiclostridium sp.KB18* |
| T36 | *Parvimonas micra* |
| T37 | *Lachnoclostridium phytofermentans* |
| T38 | *Enterococcus faecalis* |
| T39 | *Christensenella massiliensis* |
| T40 | *Libanicoccu massiliensis* |
| T41 | *Bacteroides uniformis* |
| T42 | *Fusobacterium nucleatum* |
| T43 | *Campylobacter jejuni* |
| T44 | *Ruminococcaceae bacterium CPB6* |
| T45 | *Shigella sonnei* |
| T46 | *Ethanoligenens harbinense* |
| T47 | *Streptococcus suis* |
| T48 | *Lactobacillus gasseri* |
| T49 | *Streptococcus lutetiensis* |
| T50 | *Ruminococcus albus* |
| Associated taxa identifier for Figure 4. Taxonomic level of species. | |
