# Supplementary figures and images for: Disrupted Circadian Rest-Activity Cycles in Inflammatory Bowel Disease Are Associated With Aggressive Disease Phenotype, Subclinical Inflammation, and Dysbiosis
Source: Front Med (Lausanne). 2022 Feb 4;8:770491. doi: 10.3389/fmed.2021.770491 (PMC8900134; doi:10.3389/fmed.2021.770491)

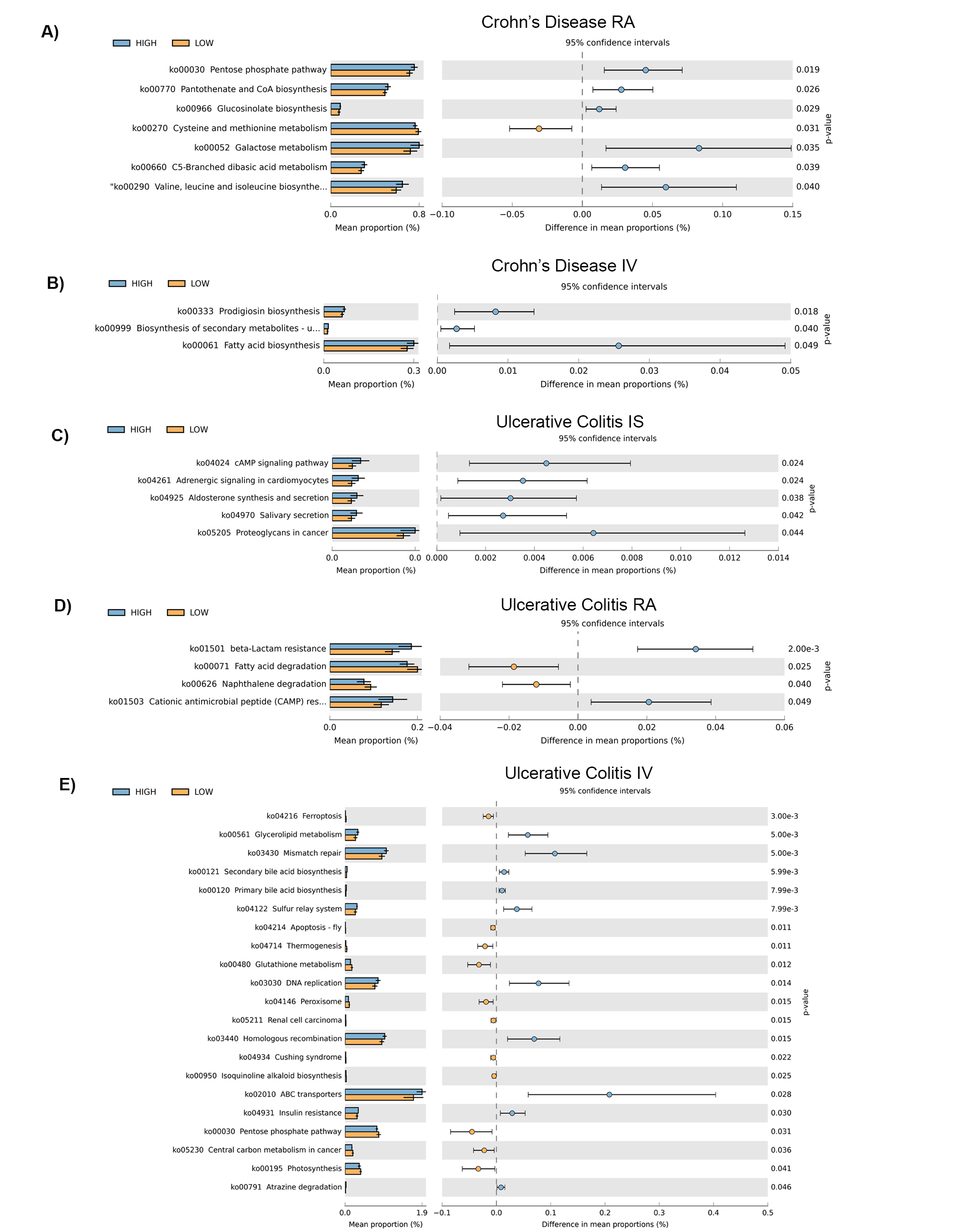

Supplement: Supplementary file 2 [file Image_1.tif]
